# METAL ION RELEASE ACCORDING TO LEG LENGTH DISCREPANCY IN CERAMIC-ON-METAL HIP ARTHROPLASTY

**DOI:** 10.1590/1413-785220233102e265272

**Published:** 2023-06-09

**Authors:** YOUNG-HO ROH, TAEHAN KANG, CHAEMOON LIM, KWANG WOO NAM

**Affiliations:** 1. Jeju National University Hospital, Department of Orthopaedic Surgery, 15 Aran 13-gil, Jeju-si, Jeju-do, South Korea.; 2 Eulji University School of Medicine, Department of Orthopaedic Surgery, Uijeongbu Eulji University Hospital, Uijeongbu-si, Gyeonggi-do, South Korea; Uijeongbu Eulji University Hospital, Uijeongbu-si, Gyeonggi-do,, South Korea

**Keywords:** Leg length inequality, Ions, Surgical procedures, operative, Follow-Up Studies, Arthroplasty, replacement, hip, Desigualdade de membros inferiores, Íons, Procedimentos cirúrgicos operatórios, Seguimentos, Artroplastia de quadril

## Abstract

**Objective:**

The ceramic-on-metal (CoM) bearing has the theoretical advantages over ceramic-on-ceramic (CoC) and metal-on-metal bearings. This study aimed to analyze factors affecting the metal ion release of CoM bearings and compare clinical performance with CoC bearings.

**Methods:**

The 147 patients were divided into 96 patients in group 1 (CoM group) and 51 patients in group 2 (CoC group). Additionally, within group1, 48 patients and 30 patients were sub-categorized into group 1-A with leg length discrepancy (LLD) less than 1cm and group 1-B greater than 1 cm. The level of serum metal ions, functional scores and plain radiographs were obtained for the analysis.

**Results:**

The level of cobalt (Co) 2-years after surgery and chromium (Cr) 1-year after surgery showed significantly higher in the group1 than the group2. LLD indicated statistically significant positive correlation between serum metal ion levels among CoM bearing THAs. In comparison of the average metal ions level changes, group 1-B showed higher level of metal ion than group 1-A.

**Conclusion:**

In patients underwent THA with CoM bearings, large LLD have a higher risk of complications associated to metal ions. Therefore, it is critical to reduce the LLD to 1 cm or less in using CoM bearing. Level of Evidence III; Case Control Study.

## RESUMO

**Objetivo:**

Uma superfície metalocerâmica (CoM) apresenta vantagens teóricas sobre as superfícies cerâmica-cerâmica (CoC) e metal-metal. Este estudo teve como objetivo analisar os fatores que afetam a liberação de íons metálicos das superfícies CoM e comparar o desempenho clínico com as superfícies CoC.

**Métodos:**

Os 147 pacientes foram divididos em 96 pacientes no grupo 1 (grupo CoM) e 51 pacientes no grupo 2 (grupo CoC). No grupo 1, 48 pacientes foram subcategorizados em grupo 1-A, com discrepância de comprimento das pernas (LLD) menor que 1 cm; e 30 pacientes no grupo1-B maior que 1 cm. O nível de íons metálicos séricos, escores funcionais e radiografias foram obtidas para a análise.

**Resultados:**

Os níveis de cobalto (Co) 2 anos após a cirurgia e de cromo (Cr), após o primeiro ano da cirurgia mostraram-se significativamente mais altos no grupo 1 do que no grupo 2. A LLD indicou correlação positiva estatisticamente significativa entre os níveis de íons do soro metálico entre os portadores de THA de CoM. Em comparação com as alterações médias dos níveis de íons metálicos, o grupo 1-B revelou um nível de íons metálicos mais alto do que o grupo 1-A.

**Conclusão:**

Em pacientes submetidos a THA com superfícies CoM e elevada LLD têm um maior risco de complicações associadas a íons metálicos. Sendo fundamental reduzir LLD para 1 cm ou menos no uso de superfícies CoM. Nível de Evidência III; Estudo de Controle de Caso.

## INTRODUCTION

Over the last decade, the increasing demand for total hip arthroplasty (THA) in younger, healthier, and high activity patients has led to the use of hard-on-hard bearing surface. In order to improve a long-term survival of THA, hard-on-hard bearings, such as ceramic-on-ceramic (CoC) and metal-on-metal (MoM), are often used. Despite its lower rate of wear and osteolysis, ceramic breakage due to brittleness of CoC bearing has been often reported.^1^ Several studies have reported that CoC bearing produce 0.004% head fracture and 0.22% liner fracture.^2^ On the other hand, main concerns for MoM bearings are metal ion release such, as cobalt (Co) and chromium (Cr), and their potential interactions with immune system leading to local reactions, such as pseudotumor and aseptic lymphocyte dominated vasculitis as well as systemic adverse effects on cardiovascular, nervous, and endocrine systems due to massive wear.^3^

To minimize complications associated with CoC and MoM bearings, a mixture of different hard bearing surfaces has created a novel option, such as the ceramic-on-metal (CoM) bearing, where a ceramic femoral head articulates with a metal alloy liner. The theoretical advantage of such combination is that it can be considered in patients with high physical activity needs due to a lower risk of component breakage compared to CoC bearings and reduced acetabular wear and metal debris production compared with MoM bearings.

Several in vitro hip simulator analyses have been conducted to investigate the wear rate of CoM bearings. Affatato et al.^4^ and Reinders et al.^5^ reported significantly greater wear in CoM bearings compared with CoC bearings. Despite benefits of in vitro study in understanding wear behaviors of each THA design, only few studies investigated the in vivo performance of CoM bearings by comparing serum metal ion levels and quantitative clinical scores between CoM and MoM bearings.

Hence, the objective of this study was to analyze factors that affect the metal ion release of the CoM bearing and to compare clinical performance with CoC bearings using validated functional outcome scores and complications.

## MATERIALS AND METHODS

### Patients

This study was a retrospective, case-control study, in which a total of 173 primary THAs and 147 patients were enrolled into the study. THA was performed using 114 CoM bearings in 96 patients and 59 CoC bearings in 51 patients. All these surgeries were performed by 1 skilled orthopedic surgeon in a single institution in the same manner. In the group 1 (CoM group), 18 patients underwent bilateral THAs, while the remaining 78 patients underwent unilateral THAs. In the group 2 (CoC group), 8 patients and 43 patients underwent bilateral and unilateral THAs, respectively. Vincent et al.^6^ reported that less than 1cm of postoperative leg length discrepancy is acceptable in THA. Among the unilateral CoM THA group, the 2 groups were divided based on leg length discrepancy (LLD) before and after surgery. 48 patients with LLD increased by less than 1cm were classified into group 1-A, and 30 patients with LLD increased by more than 1cm were classified into group 1-B. All patients undergoing THA between March 2010 and December 2015.

The inclusion criteria were as follows: (1) patients aged over 20 years; (2) patients with primary and secondary osteoarthritis, hip degeneration after previous septic arthritis, femoral neck fracture and osteonecrosis of femoral head; (3) Follow-up for at least 3 years after surgery.

**Figure 1:**
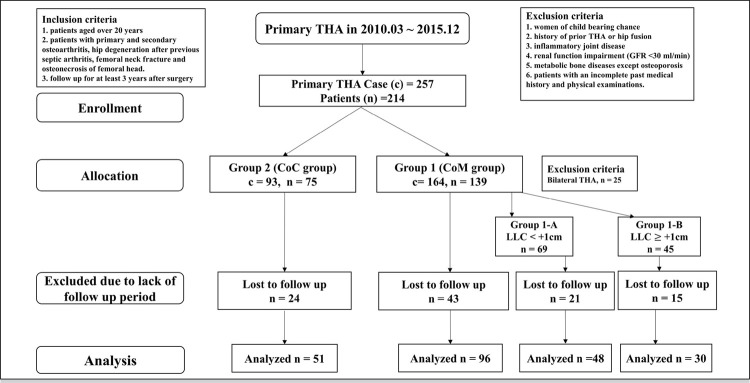
Patient enrollment flow diagram. This study involved 147 THA patients.

The exclusion criteria were as follows: (1) women of child bearing chance; (2) history of prior THA or hip fusion (3) inflammatory joint disease; (4) renal function impairment (glomerular filtration rate <30 ml/min); (5) metabolic bone diseases except osteoporosis; (6) patients with an incomplete past medical history and physical examinations. Serum metal ion levels, clinical functional outcomes, and surgical parameters were evaluated in all patients. The patient sex, age, body mass index (BMI), follow-up periods and preoperative diagnoses were investigated as demographic factors. A flow diagram for excluded cases is shown in Figure 1 .

Institutional Review Board Statement: The study was conducted in accordance with the Declaration of Helsinki and approved by the Institutional Review Board of Jeju National University Hospital (IRB No. 2015-06-010 and date of approval Aug 03, 2015).

Informed Consent Statement: Patient consent was waived because this was a retrospective and observational study with routine treatment.

### Surgical Technique and implant

All patients received identical devices in each group except only for acetabular liner (metal or ceramic). Pinnacle acetabular shell consist of Titanium-6Aluminium-4Vanadium alloy (Pinnacle; DePuy®, Warsaw, IN, USA), Biolox delta femoral head consist of Zirconia toughened Alumina composite and cementless collarless femoral stem consist of Titanium-6Aluminium-4Vanadium alloy (Summit; DePuy®, Warsaw, IN, USA) were used in all of the patients. The group 1 used the Cobalt-Chromium-Molybdenum alloy (Ultamet; DePuy®, Warsaw, IN, USA) as the acetabular metal liner and group 2 used Biolox delta liners consisting of Zirconia-Alumina as a ceramic liner.

All operations were carried out by a single orthopedic surgeon (NGW), and the surgical techniques used for the procedure were identical for all patients via posterior approach. Standard postoperative care and rehabilitation was carried out for all THA patients in the same manner. The patients were followed up in outpatient clinics at 1 month, 3 months, 6 months, 1 year and annually after the operation.

### Metal ion level measurements

Blood sampling for the metal ion analysis was obtained from all patients at 3 months, 6 months, 1 year and annually after the operation. The sample was obtained using a stainless-steel hypodermic needle attached to a plastic collecting tube, needles and collecting tubes were from the same batches. The serum Co and Cr levels were measured using a plasma mass absorption spectrometry (Spectr AA-800H, Varian Inc., Palo Alto, California, USA). The limits of detection Co and Cr were 0.05µg/L and 0.1µg/L, respectively. The toxic cut-off levels for Co and Cr were 7.00 µg/L and 7.00 µg/L or above, respectively.^7^ In CoC bearings, Co and Cr ion measurement was measured for a quality control. The Co and Cr change (∆Co and ∆Cr) was the calculation of the difference between levels of serum Co and Cr just before and 3 years after surgery. Any changes in serum ion levels were marked + for an increase and – for a decrease. All laboratory analyses were performed by personnel who were blinded to the protocol.

### Clinical outcome measurements

All patients were evaluated for clinical outcomes using the Harris Hip Score (HHS)^8^ and Western Ontario McMaster Universities Osteoarthritis Index (WOMAC)^9^ preoperatively and at 3 months, 6 months, 1 year and annually after the operation. To identify the squeaking, we performed physical examination and history taking about friction sound during the outpatient clinic visit. The research nurse completed the questionnaire directly to the patients who visited outpatient clinic by a blind protocol.

### Radiologic outcome measurements

Radiological assessment was undertaken on preoperatively, immediately postoperatively, at 6 weeks, 3 months, 6 months, 1 year, 2 years postoperatively and annually follow-up. Acetabular component inclination was recorded by measuring the angle between a horizontal inter-ischial line and a line drawn across the mouth of the component at the widest projection of the ellipse on a long-standing radiograph of the entire lower extremity. Acetabular component anteversion was measured as the angle formed by the long axis of the ellipsoid projection of the cup base and a vertical line on translateral radiographs.^10^ Assessment on postoperative radiographic measurements of leg length as defined by Paley et al.^11^ used long-standing radiographs of the entire lower extremity. The images were taken at the point when patients were stand on full weight bearing after the operation. Any LLDs were marked + for an increase and – for a decrease. For interobserver agreement of the LLD was assessed by two independent orthopedic surgeons (RYH and CML). The kappa value was 0.78, indicating substantial agreement according to the Landis and Koch criteria.^12^

### Statistics

The Clinical characteristics and surgical factors for continuous variables between the group 1 and 2 were analyzed using the Student t-test and Mann-Whitney test. In contrast, the Chi-square test and Fisher’s exact test were used for categorical variable analysis. Pearson’s correlation analysis method was used to determine the relationship between blood metal ion level and patient-related factors, surgical factors, clinical scores and location of acetabular component positions. A linear mixing model was used because there was an omission value of the measured serum metal ion level as a method to identify the change in blood metal ion levels repeated after surgery. Statistical analysis was performed using the SPSS Statistics version20.0 (IBM Corp., Armonk, NY). Statistical significance was defined as a *p* value < 0.05.

## RESULTS

A total of 173 cases and 147 patients were analyzed. There were 59 male and 88 female patients with the mean age of 61.4±١٤.6 years. The mean of the follow-up period was 6.99 ±1.36 years. The preoperative diagnosis was avascular necrosis of the femoral head in 88 cases (49.1%), proximal femoral fractures in 49 cases (29.3%), primary osteoarthritis in 24 cases (14.4%), secondary osteoarthritis in 9 cases (5.4%) and previous septic arthritis in 3 cases (1.8%). Group 1 had 114 cases, including 96 patients, and group 2 had 59 cases, including 51 patients. No differences were observed with gender ( *p* = 0.243), age ( *p* = 0.756), BMI ( *p* = 0.588) and follow-up periods ( *p* = 0.142) between the group 1 and 2. ( Table 1 )

**Table 1:** Demographic and surgical characteristics of the patients for each group.

Characteristic	Group 1 (CoM^a^; N=96)	Group 2 (CoC^b^; N=51)	p value
**Gender {Patients (hips)}**			**0.243**
Male	41 (50)	18 (22)	
Female	55 (64)	33 (37)	
**Age**			
Mean (range)	61.0 (25-90)	61.8 (35-85)	0.756
**BMI^c^**			
Mean (range)	24.3 (15.8-39.1)	24.7 (17.7-49.0)	0.588
**Diagnosis {Patients (hips)}**			
AVN^d^	44 (57)	24 (31)	
Primary OA^e^	13 (16)	8 (8)	
Secondary OA^e^	5 (5)	3 (4)	
Trauma	33 (35)	14 (14)	
Previous septic arthritis	1 (1)	2 (2)	
**Follow-up (years)**			
Mean (range)	5.1 (3.7-7.0)	4.7 (3.4-7.1)	0.142
**LLD^f^ (mm)**			
Mean (range)	6.1 (-1.1-23.3)	5.3 (-1.3-17.1)	0.348
**Acetabular cup inclination**			
Mean (range)	41.0 (25.9-55.0)	42.3 (25.7-.54.4)	0.101
**Acetabular cup anteversion**			
Mean (range)	23.3 (8.2-38.7)	23.6 (6.2-35.2)	0.755
**Cup size**			
Mean (range)	50.5 (44-60)	49.3 (44-56)	0.270
**Head size**			
Mean (range)	34.5 (28-36)	33.5 (28-36)	0.113
**Operation time (minute)**			
Mean (range)	91.0(61-229)	105.6 (74-259)	0.047*
**IntraOP^g^ Bleeding (ml)**			
Mean (range)	864.7 (150-4500)	836.6 (150-2500)	0.760

a: ceramic-on-metal, b: ceramic-on-ceramic, c: body mass index, d: avascular necrosis, e: osteoarthritis, f: leg length discrepancy, g: intraoperative. * Statistically significant.

Although the surgical characteristics of each group were similar, the mean operation time was longer ( *p* = 0.047) in the group 2. No differences were observed with LLD ( *p* = 0.348), acetabular cup inclination ( *p* = 0.101), acetabular cup anteversion ( *p* = 0.755), cup size ( *p* = 0.270), head size ( *p* = 0.113) and intra-operative bleeding ( *p* = 0.760) between group 1 and 2. ( Table 1 )

**Figure 2:**
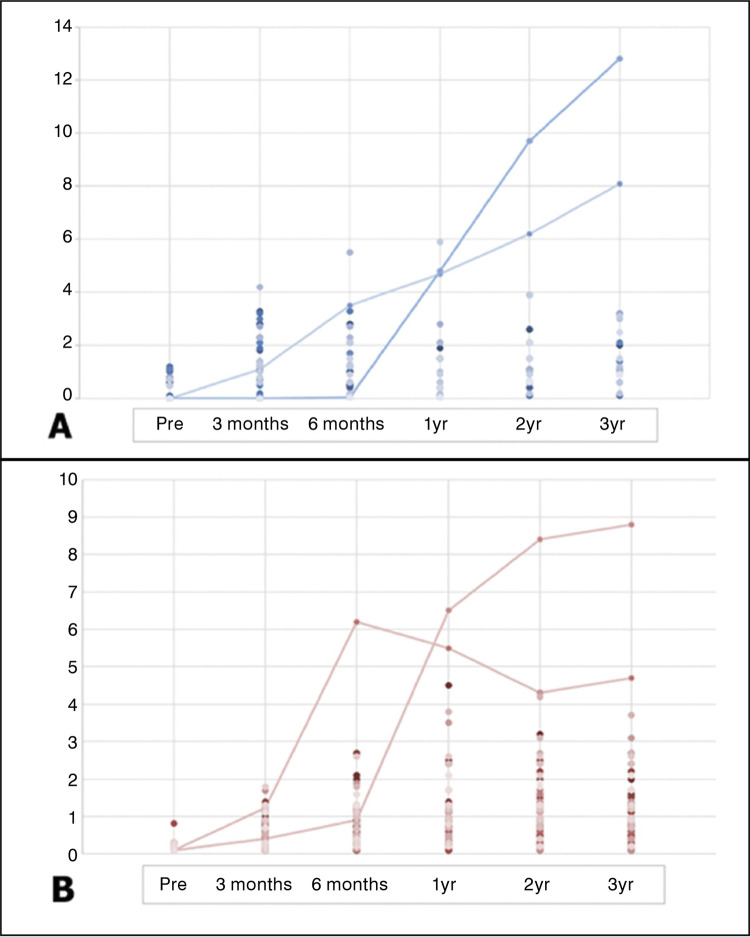
(A) Serum cobalt ion levels of each patient (µg/L; blue line: revisional arthroplasty patients) (B) Serum chromium ion levels of each patients (µg/L; red line: revisional arthroplasty patients).

When Co and Cr serum ion levels were continually examined over time, there was no significant difference between the 2 subgroups until postoperative 1 year for Co and postoperative 6 months for Cr. However, since then, there have been statistically significant changes in serum Co and Cr levels between the 2 groups. In the group 1, serum cobalt ion level measured at 2 years ( *p* = 0.041) and 3 years ( *p* = 0.026) after surgery were higher than in the group 2. In the group 1, serum Cr ion levels measured at 1 year after surgery ( *p* < 0.001), 2 years ( *p* < 0.001), and 3 years ( *p* = 0.001) were higher than in the group 2. It is shown in Figure 2A, B and Table 2 that the serum metal ion level is plotted according to the time of examination. The linear mixed model was used to analyze the interaction between time and the serum metal ion level repeatedly measured in the group 1 and 2. The results were statistically significant at both the Co ( *p* = 0.017) and Cr ( *p* < 0.001) serum ion levels.

**Table 2:** Comparison of Serum ion levels at each time point and functional scores between the 2 subgroups.

Group		Group 1 (CoM^a^; N=96)	Group 2 (CoC^b^; N=51)	p value
Metal ion (µg /L) Cobalt (range)	**Time point**			
	pre	0.22±0.32 (0.00-1.20)	0.14±0.45 (0.00-1.60)	0.874
	3 months	0.56±0.97 (0.00-4.20)	0.32±0.78 (0.00-2.10)	0.230
	6 months	0.47±1.05 (0.05-5.50)	0.50±1.19 (0.00-3.60)	0.920
	1 year	0.59±1.43 (0.05-6.90)	0.30±0.50 (0.05-2.10)	0.213
	2 years	1.38±2.56 (0.65-9.70)	0.24±0.70 (0.05-2.90)	0.041*
	3 years	1.49±1.59 (0.90-12.80)	0.29±0.81 (0.00-2.10)	0.026*
Chromium (range)	pre	0.27±0.13 (0.00-0.82)	0.25±0.19 (0.00-0.63)	0.792
	3 months	0.43±0.40 (0.00-1.80)	0.31±0.38 (0.00-1.81)	0.352
	6 months	0.67±0.90 (0.10-6.20)	0.37±0.53 (0.10-2.70)	0.096
	1 year	1.47±1.47 (0.10-6.50)	0.41±0.30 (0.10-1.98)	< 0.001*
	2 years	2.02±2.09 (0.10-8.40)	0.28±0.22 (0.10-1.90)	< 0.001*
	3 years	1.76±1.92 (0.10-8.80)	0.26±0.21 (0.10-1.45)	0.001*
**WOMAC^c^**				
Pain (0-20)		2.63±3.43 (0.10-8.80)	1.13±2.72 (0.10-8.80)	0.131
Stiffness (0-8)		0.82±1.37 (0.10-8.80)	0.74±0.74 (0.10-8.80)	0.065
Function (0-68)		12.89±13.70 (0.10-8.80)	11.85±9.53 (0.10-8.80)	0.054
Total (0-96)		12.34±17.49 (0.10-8.80)	10.45±11.35 (0.10-8.80)	0.083
HHS^d^ (0-100)		83.80±9.85 (61-96)	87.76±7.06 (72-96)	0.159

a: ceramic-on-metal, b: ceramic-on-ceramic, c: Western Ontario and McMaster Universities Osteoarthritis Index, d: arris Hip Score. Values are expressed as mean ± standard deviation (range, µg/L in metal ion). * Statistically significant.

Clinical outcomes of group 1 and 2 were analyzed based on the results of the most recent examination. No statistical differences in pain ( *p* = 0.131), stiffness ( *p* = 0.065), function ( *p* = 0.054) and total WOMAC scores ( *p* = 0.083). Similarly, no significant differences were seen between the 2 groups in HHS ( *p* = 0.159) ( Table 2 ). The squeaking incidence were observed in 15 cases in the group 1 (15.6%) and in 6 cases in the group 2 (11.1%). There was no statistically significant difference in friction incidence between group 1 and 2. ( *p* = 0.358).

Correlations among last follow-up serum metal ion level, patient related factors, surgical characteristics, acetabular component positions and clinical outcomes were assessed in CoM THA patients. There were statistically significant positive correlations between serum Co levels and LLD ( *r* = 0.211, *p* = 0.046) as well as between the serum Cr levels and LLD ( *r* = 0.281, *p* = 0.007). In addition, surgical characteristics, acetabular cup position, head and cup size did not show any correlations with the metal ion level ( Table 3 ). In comparison of average changes in metal ion levels, group 1-B (0.25 ± 0.77 µg/L) showed more increases than group 1-A (0.13 ± 0.31 µg/L) in Co ion, but it did not have statistical significance ( *p* = 0.055). Also, group 1-B (0.68 ± 0.77 µg/L) showed higher increases in Cr ion level changes than group 1-A (0.34 ± 0.34 µg/L) with statistical significance ( *p* = 0.025). ( Table 3 )

**Table 3:** Correlation between each variable and serum metal ion levels in the patients with CoM group. Comparison of serum metal ion levels change in unilateral CoM group, according to the difference of leg length after surgery.

CoM^a^ group Factor	Cobalt	P value	Chromium	p value
Age	r = -0.056	0.602	r = 0.034	0.753
BMI^b^	r = 0.075	0.481	r = 0.079	0.458
Op.^c^ time	r = -0.002	0.984	r = 0.047	0.659
Op.^c^ Bleeding	r = -0.021	0.843	r = -0.025	0.816
AI^d^	r = 0.189	0.074	r = 0.126	0.236
AA^e^	r = 0.172	0.070	r = 0.182	0.074
LLD^f^	r = 0.211	0.046*	r = 0.281	0.007*
Head size	r = 0.107	0.316	r = 0.096	0.367
Cup size	r = -0.030	0.777	r = 0.041	0.698
HHS^g^	r = 0.135	0.405	r = 0.117	0.471
Pain	r = 0.019	0.868	r = 0.110	0.342
Stiffness	r = 0.004	0.971	r = 0.104	0.370
Function	r = -0.009	0.937	r = 0.028	0.811
Total WOMAC^h^	r = -0.003	0.979	r = 0.052	0.657
**CoM^a^ group (N = 78)**			**p value**	
**Metal ion (µg /L)**	**Group 1-A^i^ (N = 48)**	**Group 1-B^j^ (N = 30)**		
∆Co^k^	0.13±0.31	0.25±0.77	0.055	
∆Cr^l^	0.34±0.34	0.68±0.76	0.025*	

a: ceramic-on-metal, b: body mass index, c: operation, d: acetabular cup inclination, e: acetabular cup anteversion, f: leg length discrepancy, g: Harris Hip Score, h: Western Ontario and McMaster Universities Osteoarthritis Index, i: LLD < +1cm, j: LLD ≥ +1 cm, k: serum cobalt ion level change, l: serum chromium ion level change. r, Pearson’s coefficient of correlation. * Statistically significant.

There were 2 patients in the group 1 with adverse local reactions to metal debris (ARMD) with metal liner wear. Revision arthroplasty was performed with CoC bearing to improve painful symptoms and prevent systemic adverse reactions induced by metal ions debris. Furthermore, other complications include periprosthetic fracture in greater trochanter, which was treated with internal fixation, and postoperative wound infections, which was controlled after simple wound irrigation and debridement.

## DISCUSSION

MoM bearing is relatively vulnerable to wear, and CoC bearing has risks of breakage that would require revision operation even though its occurrence is relatively lower. Consequently, CoM bearings has been proposed as a durable alternative treatment option for patients with high physical activity needs.

The use of CoM bearing is advantageous in expansion in the selection of various sizes of ceramic heads even in a small acetabular cup. In our study, most pateints were performed with THAs using head sizes less than diameters of 36mm, and there is report on statistically lower rates of dislocation on head diameters of 36mm than 28mm. It can theoretically reduce dislocation and increase ROM compared to other bearings.^13^ However, Han et al.^14^ ironically concluded that the large ROM is the factor that increases the metal ion levels. In addition, there were studies that differential hardness bearing improves fluid film lubrication and reduces adhesive wear and that in vitro studies have proven that differential hardness through a CoM bearing avoids stripe wear.^15^

In several previous biomechanical and clinical studies, CoM bearing is reported to reduce the metal wear and metal ion release compared to the MoM bearing.^16^ However, the CoM bearings showed the results over a wide range of performance in vivo studies. Affatato et al.^4^ reported more wears occurred in the CoM bearing than in the CoC bearing, and Reinders et al.^5^ no differences in mean wear rates between CoM and CoC bearings.

In this study, the serum ion levels of Co ( *p* = 0.026) and Cr ( *p* = 0.001) at 3 years postoperatively were significantly higher in the group 1 than in the group 2. Han et al.^14^ demonstrated that serum metal ion levels in the CoM bearings were a 6.5-fold and 9-fold higher in the Co and Cr, respectively, in comparison to non-CoM bearings. In this study, the metal ion levels in the group 1 showed a 5.1-fold higher level of Co and a 6.8-fold higher level of Cr than those in the group 2.

The patterns of metal ion changes in the 2 subgroups were noted in relation to time with statistical significances (Co: *p* = 0.017; Cr: *p* < 0.001) and showed gradual increases with the number of cycles similar to previous studies.^4 , 5^ It has increased to a similar level to other studies^14^ only to the extent without the toxic effect.^7^ Even though the serum metal ion level did not show any correlations with other variables like age, BMI, surgical factor, acetabular cup position and clinical outcomes, LLD showed positive correlations with both Co (r = 0.211, *p* = 0.046) and Cr (r = 0.281, *p* = 0.007) serum ion levels. In addition, group 1-B indicated larger serum ion level changes in Co and Cr than group 1-A, and the increase of Cr level in group 1-B showed statistical significance ( *p* = 0.025). These results suggest that LLD may affect the functional position of the cup and the edge load. A recent study by Renkawitz et al.^17^ reported the occurrence of unphysiological gait on patient with more than 5mm leg length difference after THAs. Other studies of risk factors for metal ion releases in THA with CoM bearing suggested BMI and amount of anteversion,^18^ but no association was found in our study.

**Figure 3:**
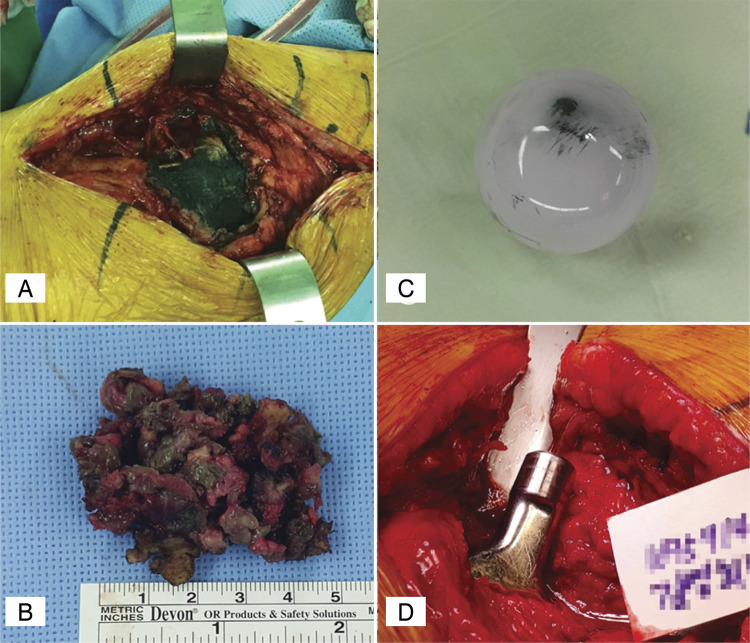
(A) Massive metal debri and adverse local reactions to metal debris were observed around the hip joint (B) Massive metal debri and metal staining of soft tissue were observed (C) Metal debri staining was observed on the ceramic head by metal liner wear (D) Taper of the stem was relatively clean and had few scratches.

There have been few direct comparisons of clinical results with the CoM and CoC bearing. As the squeaking incidence of about 10% reported in the previous study,^19^ the squeaking incidence of CoC and CoM bearing in our study was 11.1% and 15.6%, respectively. In addition, the difference in the squeaking rate between group 1 and 2 was not statistically significant ( *p* = 0.358). In this study, there were 2 patients in group 1 who suffered from metal liner wear along with outlier serum metal levels, and both patients received revision arthroplasty with CoC bearings. This result shows that revision rate occurred in only 2 out of 114 (1.8%) cases, which is a good result compared to other studies.^20^ Their surgical findings revealed massive metal debris with ARMD ( Figure 3A, B ) as well as metal staining in the ceramic head and severe wear at the inferior edge. The LLD were 18.7mm and 14.3mm in first and second patients with revision arthroplasty, respectively. According to the surgical findings, the ceramic head was metal stained, and the taper was relatively clean ( Figure 3C, D ). Therefore, the metal liner wear on the CoM bearing is considered to be the cause, and the possibility of edge loading due to the difference in leg length after surgery is suspected.

There are several limitations to this study. Our study was not conducted with randomization and blinding protocol in all the procedures for surgery and data collection. Therefore, explanations of all the variables utilized in our study are vulnerable to possible inherent biases. There is a problem with the mismatch that the number of patients and the gender ratio between the 2 subgroups. And although the number of patients sampled is not small, not all patients were followed-up. In addition, the study has a limitation that the confirmation of squeaking depended entirely on the patient’s answer. On the other hand, the strength of the study is that 1 skilled orthopedic surgeon operated all patients in the same manner at the 1 institution

## CONCLUSION

Our results suggest that CoM bearing THAs show effective hip function in both short-term and mid-term similar to CoC bearing THAs even though serum ion levels of Co and Cr were relatively higher in CoM group and especially higher in patients with LLD more than 1 cm. Therefore, it is critical to minimize LLD less than 1cm during CoM bearing THA operation, and in cases with postoperative LLD more than 1 cm, periodic changes of serum metal ion levels as well as possible clinical symptoms of metal ion toxicity should be carefully examined.
